# Identification of miR-203a, mir-10a, and miR-194 as predictors for risk of lymphovascular invasion in head and neck cancers

**DOI:** 10.18632/oncotarget.28022

**Published:** 2021-07-20

**Authors:** Moumita Karmakar, Pei-Chun Lai, Samiran Sinha, Shannon Glaser, Sanjukta Chakraborty

**Affiliations:** ^1^Department of Statistics, Texas A&M University, College Station, TX 77843, USA; ^2^Department of Medical Physiology, Texas A&M Health Science Center, College of Medicine, Medical Research and Education Building, Bryan, TX 77807, USA

**Keywords:** miRNA, supervised clustering, random forest, TCGA, head neck cancer

## Abstract

Lymphovascular invasion (LVI) is an important prognostic indicator of lymph node metastasis and disease aggressiveness but clear molecular mechanisms mediating this in head and neck cancers (HNSC) remain undefined. To identify important microRNAs (miRNAs) in HNSC that associate with and are also predictive of increased risk of LVI, we used a combination of clustering algorithms, multiple regression analyses and machine learning approaches and analyzed miRNA expression profiles in the TCGA HNSC database. As the first step, we identified miRNAs with increased association with LVI as a binary variable. In order to determine whether the identified miRNAs would show functional clusters that are also indicative of increased risk for LVI, we carried out unsupervised as well as supervised clustering. Our results identified distinct clusters of miRNAs that are predictive of increased LVI. We further refined these findings using a Random forest approach, and miR-203a-3p, mir-10a-5p, and miR-194-5p to be most strongly associated with LVI. Pathway enrichment analysis showed these miRNAs targeted genes involved in Hippo signaling and fatty acid oxidation pathways that are mediators of lymph node metastasis. Specific association was also identified between the miRNAs associated with LVI and expression of several lymphangiogenic genes that could be critical for determination of therapeutic strategies.

## INTRODUCTION

Recurrent or metastatic head and neck cancer (HNSC) that includes tumors of oral cavity, paranasal sinuses, nasal cavity, pharynx, and larynx is associated with poor patient outcome, tumor aggressiveness and is characterized by early metastasis to the regional lymph nodes [1]. It is the 6th most common cancer worldwide and has a 5-year survival rate of less than 50% which is one of the lowest among major cancers [2]. Recurrence of HNSC is the primary clinical event limiting success of therapeutic interventions after surgical tumor resection [3]. Additionally, patients with HNSC often have limited options therapy as tumors in this region show profound drug and chemoresistance warranting development of new therapies [4]. Integrated analysis of multi-dimensional transcriptomic data is important to our understanding of cancer metastasis and could provide valuable clues to tumor stage progression, dysregulated cellular pathways and survival outcome [4]. A high proportion of patients who do not respond to standard treatment could get a benefit from personalized therapy based on the molecular diagnostics or targeted therapies to a particular tumor grade or stage and specific patterns of dissemination [5]. Studies have established several risk factors such as tobacco use and HPV status as primary risk agents for HNSC [6]. In HNSC, metastatic dissemination to regional lymph nodes has been shown to be a major prognostic indicator for disease progression, and positive lymph node association reduces survival by 50% [7]. Lymphovascular invasion (LVI) (defined as the presence of tumor cells within a definite endothelial-lined space (lymphatics or blood vessels), is a key predictor of metastatic spread [8]. LVI of tumor cells is a prerequisite for the dissemination via the lymphatic or blood vascular system and increased lymphatic vessels density (or lymphangiogenesis) near tumor cells are more likely to promote tumor spread to lymph nodes and to distant sites [9, 10]. LVI is a histopathological feature that established as an independent predictor of poor prognosis and lymph node metastasis (LNM) in several solid tumors. Although different molecular signatures are associated with HNSCC, most studies have typically overlooked the association of any molecular, genetic or clinical features with LVI and the factors contributing to LVI remain very poorly understood [11–13].

Large scale genome sequencing projects by The Cancer Genome Atlas (TCGA) have established extensive patient datasets of HNSC with detailed information of molecular characteristics of the tumor [14]. As cancer cell behavior is governed by multiple, nonlinear, interacting pathways, integrated analysis of multidimensional transcriptomic data provides valuable clues to tumor stage progression, cell signaling and survival outcome [15]. Several biomarkers for HNSC have been defined but most of these have failed to have prognostic value for recurrent disease, possibly because of any lack of association with lymph node metastasis. Recently miRNAs, that are small non-coding RNA about 18–22 nucleotides long have emerged as significant predictors of different disease outcomes and have established their efficacy as therapeutic biomarkers [16]. miRNAs modulate specific gene expression by inhibiting specific genes or gene networks and thereby affect an entire biological pathway. Several studies have identified various miRNAs to be dysregulated in HNSC, and are shown to be involved in regulation of various molecular pathways and cellular processes that contribute to tumor progression and metastasis in HNSC [17–19]. However, the correlation between miRNA expression and regulation of genes involved in promoting LVI and subsequently metastatic disease, in particular lymph node metastasis, remains grossly understudied. There is thus a considerable knowledge gap in the application of miRNAs as potential biomarkers or prognostic indicators for LVI and thus eludes development of targeted therapeutic strategies. Further, since miRNAs target several groups of genes during cancer progression [20], identification of miRNA clusters that are closely predictive or associated with LVI maybe more clinically relevant than identifying single miRNA as they would provide critical information about specific pathways that are dysregulated during progression of LVI and subsequent metastasis.

Hence, in this paper, we address this critical gap and use sophisticated machine learning and clustering approaches to systematically identify specific clusters of miRNAs that are predictive of and show significant association with LVI and thus could be further evaluated as prognostic indicators of tumor spread in HNSCC. Further, a novel aspect of these studies was that as lymphangiogenesis or growth of new lymphatic vessels is a critical event that promotes LVI and subsequently lymph node metastasis (LNM), we also evaluated whether these miRNAs showed correlation with specific lymphangiogenic genes.

## RESULTS

Based on the 5% significance level criteria, we have selected 61 out of 496 miRNAs that are strongly associated with the LVI status. The odds ratio estimate and the 95% confidence interval for the association between LVI and the 61 miRNAs are given in Table 1.

**Table 1:** The odds ratio estimate and the 95%Wald’s confidence interval from the univariate logistic regression analyses, for all 61 miRNAs

mimiRNA.Name	OR	Lower limit (95% CI)	Upper limit (95% CI)
hsa-miR-203a-3p	0.60	0.47	0.76
hsa-miR-30a-5p	1.60	1.25	2.04
hsa-miR-30a-3p	1.58	1.25	2.01
hsa-miR-10a-5p	1.59	1.25	2.03
hsa-miR-1293	0.65	0.51	0.83
hsa-miR-30c-2-3p	1.52	1.19	1.92
hsa-let-7d-5p	1.52	1.19	1.94
hsa-miR-28-5p	1.50	1.18	1.91
hsa-miR-128-3p	1.48	1.16	1.88
hsa-miR-3913-5p	1.49	1.17	1.90
hsa-miR-1266-5p	1.46	1.16	1.84
hsa-miR-194-5p	1.44	1.14	1.82
hsa-miR-9-5p	1.42	1.13	1.79
hsa-miR-106b-5p	1.42	1.12	1.80
hsa-miR-25-3p	1.43	1.12	1.81
hsa-miR-204-5p	1.41	1.11	1.77
hsa-miR-218-5p	1.40	1.11	1.77
hsa-miR-101-3p	1.40	1.11	1.78
hsa-miR-92b-3p	1.39	1.10	1.76
hsa-miR-181c-3p	1.39	1.10	1.76
hsa-miR-31-3p	0.73	0.58	0.91
hsa-miR-192-5p	1.39	1.10	1.75
hsa-miR-1287-5p	1.39	1.10	1.76
hsa-miR-584-5p	0.72	0.57	0.91
hsa-miR-181d-5p	1.37	1.09	1.73
hsa-miR-30d-3p	1.38	1.09	1.76
hsa-miR-95-3p	1.36	1.08	1.72
hsa-miR-9-3p	1.34	1.07	1.68
hsa-miR-101-5p	1.36	1.07	1.71
hsa-miR-187-3p	0.75	0.59	0.94
hsa-miR-497-5p	1.35	1.06	1.70
hsa-miR-217-5p	1.33	1.06	1.68
hsa-miR-1910-5p	0.74	0.58	0.95
hsa-miR-130b-3p	1.33	1.05	1.68
hsa-miR-141-5p	1.33	1.05	1.69
hsa-miR-455-3p	0.75	0.60	0.95
hsa-miR-31-5p	0.76	0.61	0.95
hsa-miR-193b-5p	0.75	0.60	0.95
hsa-miR-340-5p	1.32	1.04	1.66
hsa-miR-20b-5p	1.31	1.04	1.64
hsa-miR-592	1.31	1.04	1.65
hsa-miR-28-3p	1.31	1.04	1.66
hsa-miR-374a-3p	1.32	1.04	1.68
hsa-miR-374b-5p	1.30	1.03	1.65
hsa-miR-378c	1.30	1.03	1.63
hsa-miR-34a-5p	1.30	1.03	1.64
hsa-miR-370-5p	1.29	1.03	1.63
hsa-miR-6720-3p	1.29	1.03	1.63
hsa-miR-194-3p	1.29	1.02	1.62
hsa-miR-769-5p	1.29	1.02	1.62
hsa-miR-6892-5p	0.76	0.60	0.98
hsa-miR-1304-3p	0.75	0.58	0.97
hsa-miR-374b-3p	1.28	1.01	1.61
hsa-miR-30b-5p	1.27	1.01	1.61
hsa-let-7d-3p	1.28	1.01	1.62
hsa-miR-195-3p	1.27	1.01	1.60
hsa-miR-151a-3p	1.27	1.01	1.60
hsa-miR-215-5p	1.26	1.00	1.59
hsa-miR-4677-3p	1.26	1.00	1.59
hsa-miR-1468-5p	1.26	1.00	1.59
hsa-let-7f-1-3p	1.26	1.00	1.59

### Identification of important miRNAs by univariate logistic regression analysis

**Figure 1:**
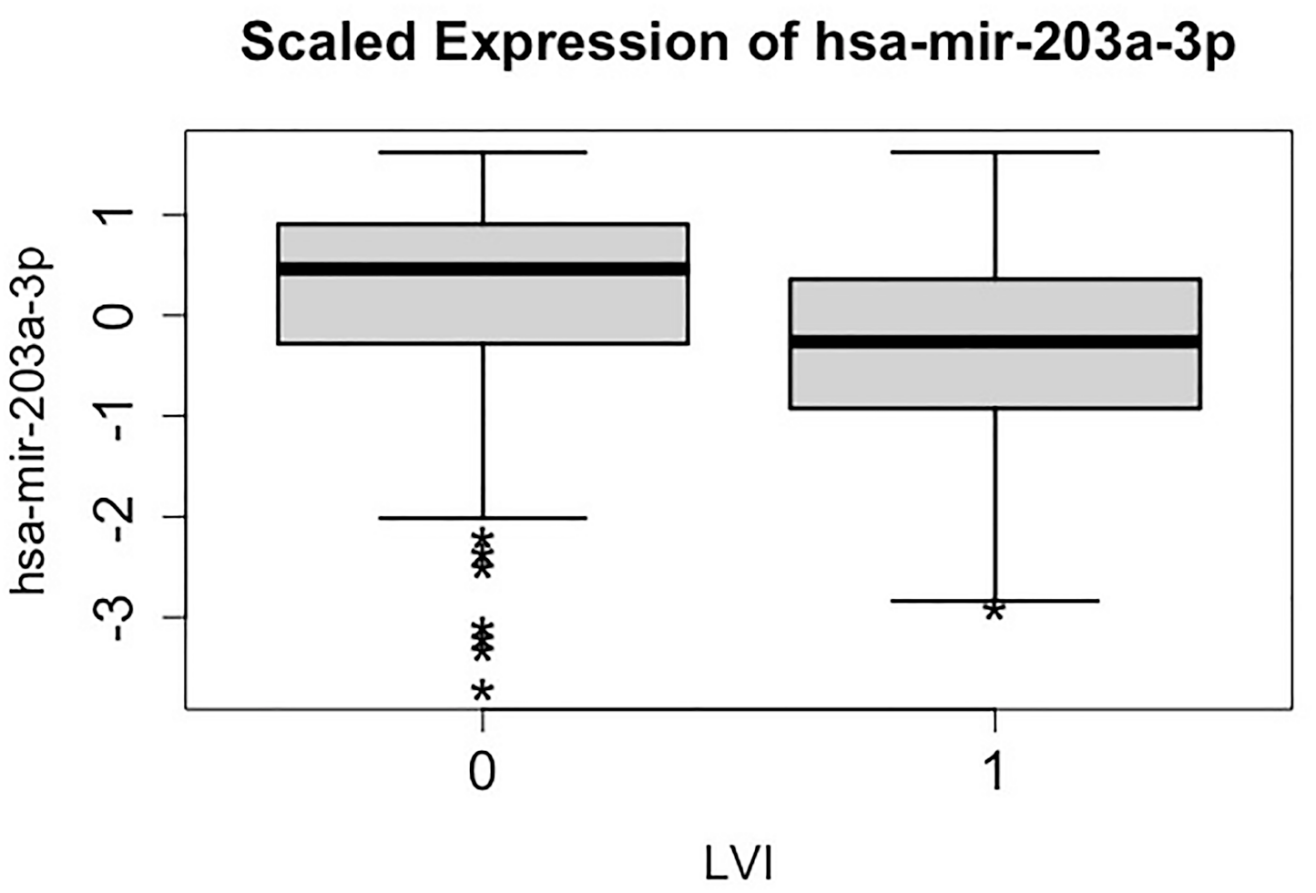
Side-by-side boxplots (distribution) of the scaled expression of hsa-mir-203a-3p against lymphovascular invasion (LVI) status. LVI value 1 and 0 indicate groups having and not having lymphovascular invasion respectively.

For the purpose of illustration here we explain the odds ratio of the most significant miRNA, hsa-miR-203a-3p, in terms of the association between the miRNA and the LVI status. The result implies that the odds of having LVI decreases by 40% for one unit increase of the scaled hsa-miR-203a-3p. This is indicated in the side-by-side boxplot as shown in Figure 1, where the distribution of hsa-miR-203a-3p for the LVI = 1 group is shifted downward compared to that for group LVI = 0.

### Pairwise correlation identifies pairs of highly correlated miRNA that associate with LVI

The pairwise correlation matrix of the 61 miRNAs reveals that hsa-miR-31-5p is highly correlated with hsa-miR-31-3p with a correlation of 0.96, hsa-miR-9-5p is highly correlated with hsa-miR-31-3p with a correlation of 0.91, hsa-miR-194-5p is highly correlated with hsa-miR-192-5p with a correlation of 0.90, and hsa-miR-30a-3p is highly correlated with hsa-miR-30c-2-3p with a correlation of 0.87. As this high correlation can cause interference to our next multiple regression analysis, we decided to remove hsa-miR-31-3p, hsa-miR-9-3p, hsa-miR-192-5p, and hsa-miR-30c-2-3p from the list of 61, resulting in 57 distinct miRNAs in the next step of analysis (data not shown).

### Identification of predictive miRNAs using logistic LASSO screening and multiple logistic regression

**Figure 2:**
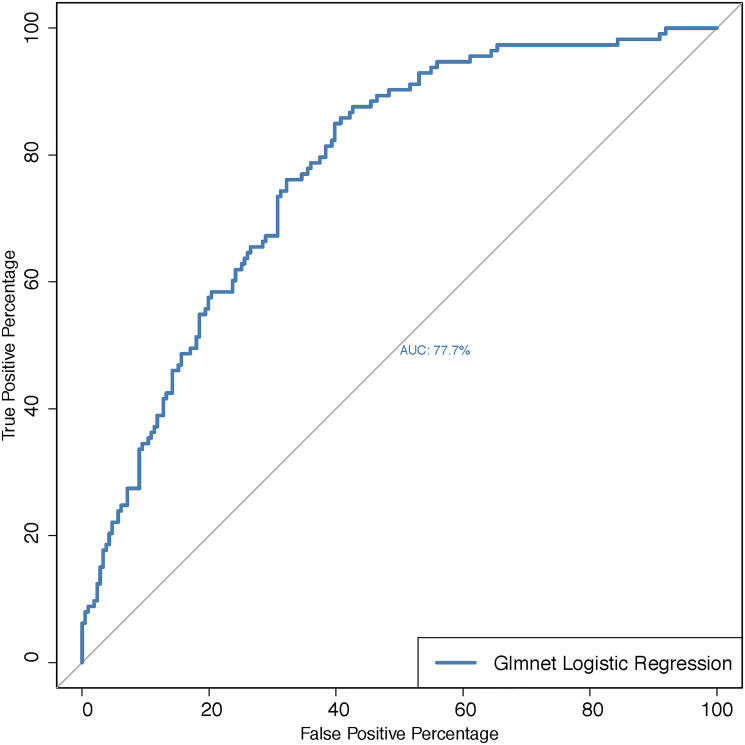
ROC-curve for predicting lymphovascular invasion status for the multiple logistic regression with the set of miRNAs as predictors that are selected via the LASSO method. Abbreviations: AUC: Area under the curve; GLMNET: Regularized Generalized Linear Model.

For identifying a predictive model for LVI, next we regressed LVI on the 57 miRNAs (after eliminating the 4 miRNAs that showed high correlation as described above). We used a multiple logistic regression method, and used the LASSO (Least Absolute Shrinkage and Selection Operator) approach to obtain regularized parameter estimates [21]. The LASSO method helps to select important features among many while sets the coefficients corresponding to unimportant features to zero. For the HNSC data, only 20 miRNAs got selected. These include hsa-let-7d-5p, hsa-miR-30a-5p, hsa-miR-30a-3p, hsa-miR-95-3p, hsa-miR-10a-5p, hsa-miR-187-3p, hsa-miR-203a-3p, hsa-miR-128-3p, hsa-miR-92b-3p, hsa-let-7f-1-3p, hsa-miR-30d-3p, hsa-miR-141-5p, hsa-miR-194-3p, hsa-miR-455-3p, hsa-miR-1910-5p, hsa-miR-3913-5p, hsa-miR-1304-3p, hsa-miR-6720-3p, hsa-miR-370-5p, hsa-miR-6892-5p. Based on these selected 20 miRNAs we ran a multiple logistic regression with LVI as the response variable. The predictive power of this multiple logistic model is presented through the ROC curve (Figure 2) with the area under the curve 77%.

### Clustering of the selected miRNAs reveals three distinct miRNA clusters that associate with LVI in HNSC

**Figure 3:**
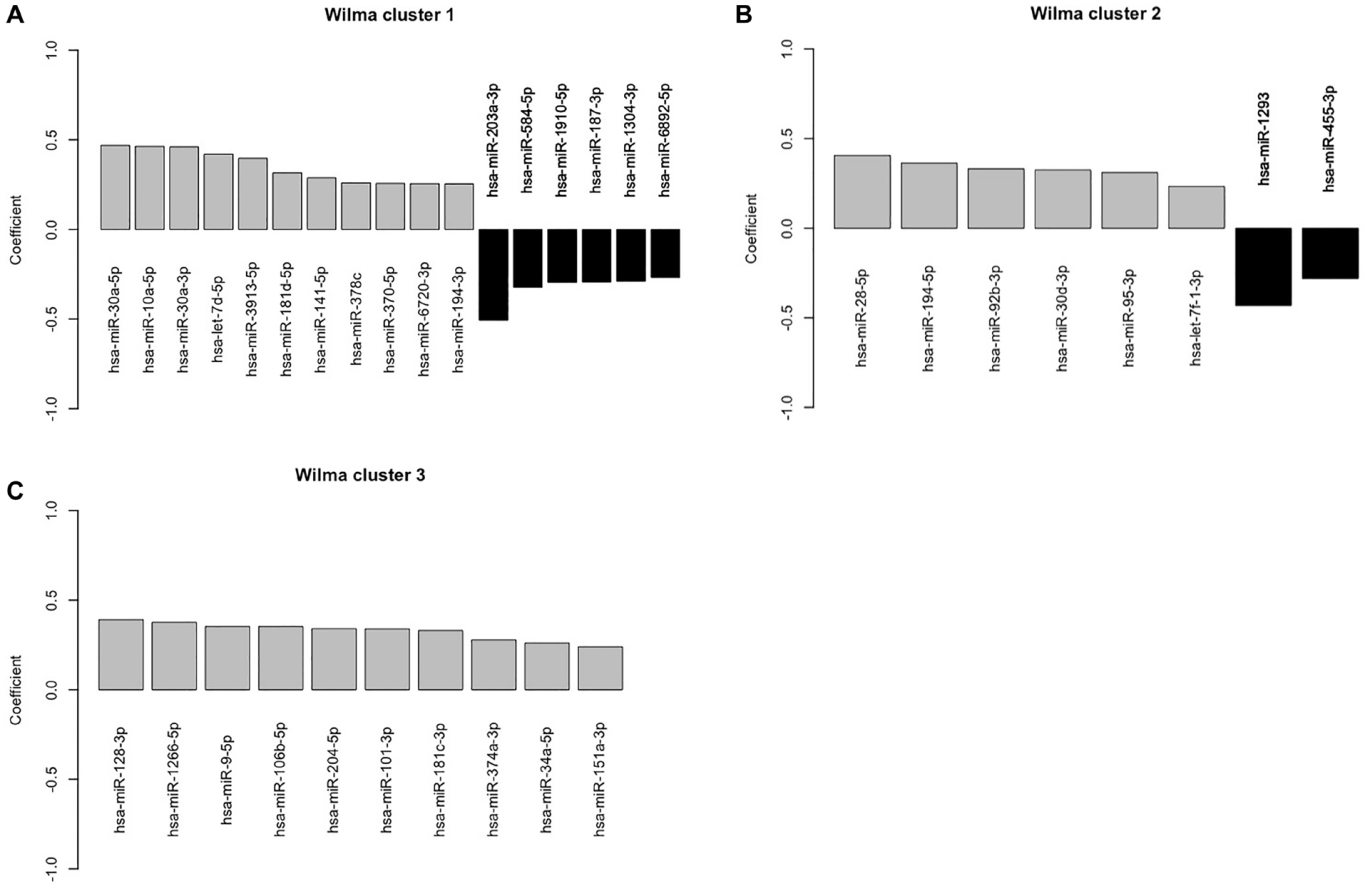
Plot of the estimated regression parameter of the univariate logistic regression for lymphovascular invasion against corresponding to each of the miRNAs that are selected by the supervised clustering method. (**A**–**C**) A positive value (grey bar) of a regression coefficient indicates that the chance of LVI increases with the expression value of the corresponding miRNAs. Similarly, a negative coefficient (black bar) indicates that the chance of LVI decreases as the expression of the corresponding miRNAs increases.

**Figure 4:**
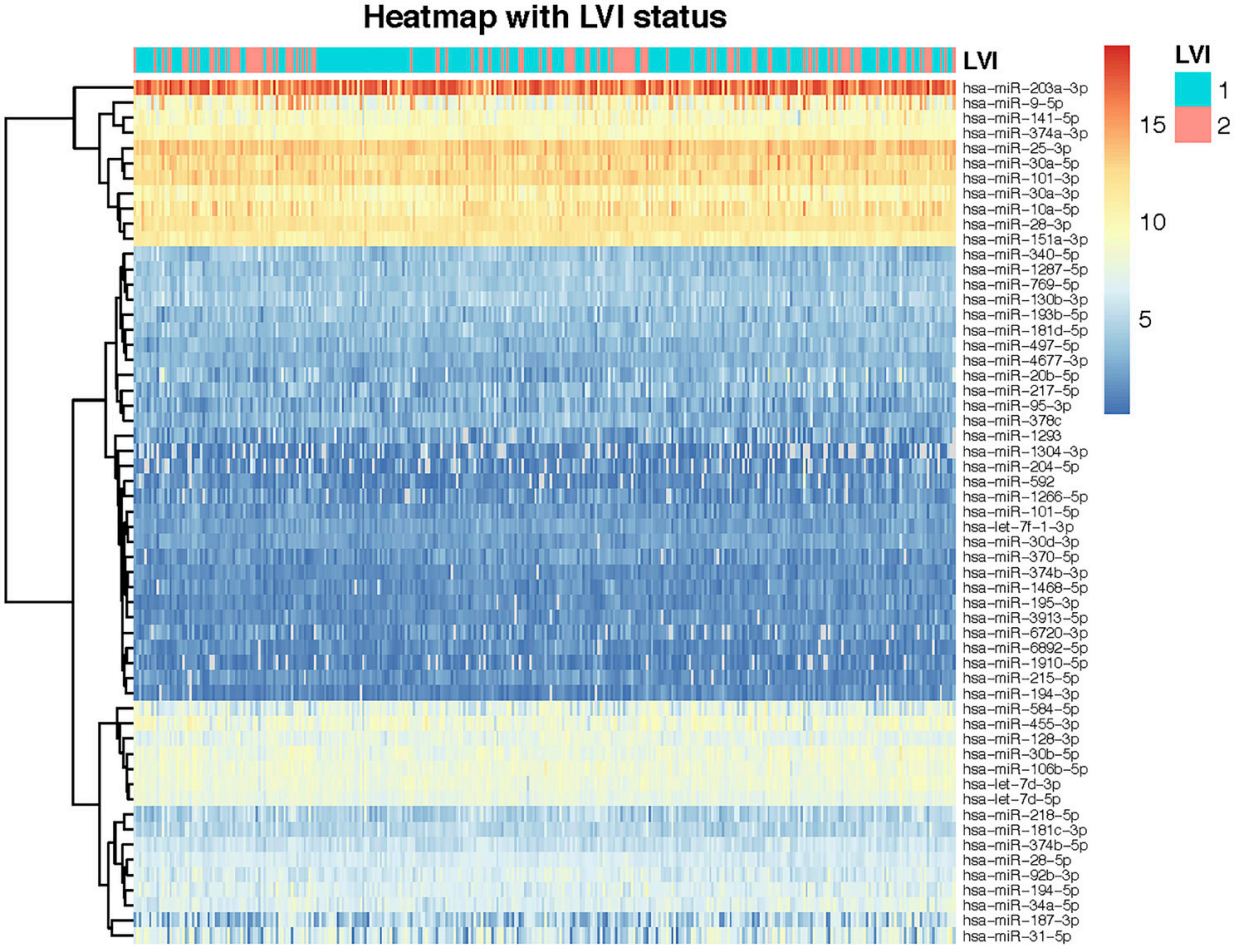
Heatmap of miRNA expressions for 324 patients showing unsupervised clustering of miRNAs. miRNAs are represented across rows while patients are represented across columns. The dendrogram depicting unsupervised clustering of miRNAs is shown on the left column of the heatmap. The LVI status of patients is shown on the top of the rows. Blue represents patients not having invasion while red represents patients that do report LVI.

Using the gap statistic, we found that the optimal number of clusters is three. Table 2 shows all the miRNAs in three unsupervised individual clusters. We then used 3 as the number of clusters in the supervised clustering method Wilma. Among the 57 miRNAs, Wilma recognized 35 miRNAs as significant for prediction of LVI status and clustered them into three different and distinct clusters. Figure 3 shows the univariate logistic regression coefficients of the miRNAs grouped into three clusters. A positive value (grey bar) of a regression coefficient indicates that the chance of LVI increases with the expression value of the corresponding miRNAs. Similarly, a negative coefficient (black bar) indicates that the chance of LVI decreases as the expression of the corresponding miRNAs increases. Cluster 1 has seventeen miRNAs, cluster 2 has eight, and cluster 3 has ten miRNAs (Figure 4). The Wilma results suggest that 35 out of 57 miRNAs are important for predicting the risk of LVI.

**Table 2:** Table of the miRNA clusters based on the K-means unsupervised approach

Cluster 1	Cluster 2	Cluster 3
hsa-let-7d-5p	hsa-miR-31-5p	hsa-miR-28-5p
hsa-miR-25-3p	hsa-miR-187-3p	hsa-miR-30a-5p
hsa-miR-34a-5p	hsa-miR-203a-3p	hsa-miR-30a-3p
hsa-miR-30b-5p	hsa-miR-584-5p	hsa-miR-95-3p
hsa-miR-9-5p	hsa-miR-193b-5p	hsa-miR-101-3p
hsa-miR-106b-5p	hsa-miR-455-3p	hsa-miR-10a-5p
hsa-miR-130b-3p	hsa-miR-1293	hsa-miR-204-5p
hsa-miR-20b-5p	hsa-miR-1910-5p	hsa-miR-215-5p
hsa-miR-592	hsa-miR-1304-3p	hsa-miR-217-5p
hsa-let-7d-3p	hsa-miR-6892-5p	hsa-miR-218-5p
hsa-miR-28-3p		hsa-miR-128-3p
hsa-miR-30d-3p		hsa-miR-194-5p
hsa-miR-141-5p		hsa-miR-151a-3p
hsa-miR-194-3p		hsa-miR-497-5p
hsa-miR-374a-3p		hsa-miR-181d-5p
hsa-miR-374b-5p		hsa-miR-92b-3p
hsa-miR-374b-3p		hsa-miR-769-5p
hsa-miR-1266-5p		hsa-let-7f-1-3p
hsa-miR-4677-3p		hsa-miR-101-5p
		hsa-miR-181c-3p
		hsa-miR-195-3p
		hsa-miR-340-5p
		hsa-miR-1287-5p
		hsa-miR-1468-5p
		hsa-miR-378c
		hsa-miR-3913-5p
		hsa-miR-6720-3p
		hsa-miR-370-5p

**Figure 5:**
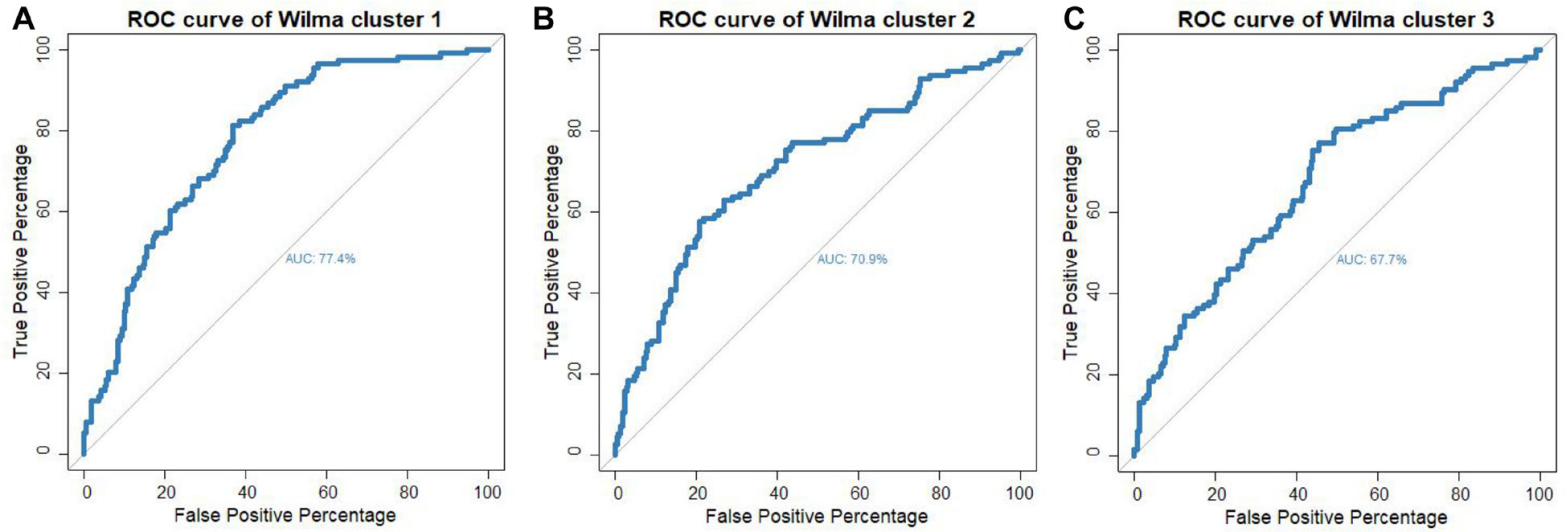
ROC-curve for predicting lymphovascular invasion status for the multiple logistic model with the set of miRNAs as predictors that are in (**A**) supervised cluster 1 (**B**) supervised cluster 2 and (**C**) supervised cluster 3.

For comparing the prediction power of each of the three supervised clusters, we fit a multiple logistic regression model for LVI on the miRNAs of each of the clusters separately. This resulted in three multiple logistic regression models and corresponding to each model we calculated the area under the ROC curve (Figure 5A–5C). Our results showed that miRNAs belonging to Cluster 1 have the highest predictive power (77%) while miRNAs in cluster 2 have the second highest predictive power (70%). It is important to note that despite a smaller size of cluster 2 compared to cluster 3, cluster 2 shows a much higher predictive performance than that of cluster 3. Further, it is important to note that the predictive power of cluster 1 is the same as the predictive power of the multiple logistic regression model where all 57 miRNAs were included as predictors (Figure 2).

Further, an unsupervised hierarchical clustering of the 57 miRNAs were also represented as a heatmap along with the LVI status (Yes or No) (Figure 4). Both the k-means and hierarchical clustering seem to indicate that there are three major clusters (Figures 4 and 9).

### Random forest analysis identifies hsa-miR-203a-3p, hsa-mir-10a-5p, hsa-miR-194-5p to be strongly associated with LVI

Further, to determine the most important miRNAs out of these 35 miRNAs that are identified by the Wilma cluster, we performed a random forest (RF) analysis as described earlier [22]. In the RF analysis, we used LVI as the binary response variable and used all 35 miRNAs as potential predictors. We first randomly split the data of 324 subjects into training and test sets consisting of 80% and 20% data, respectively. This resulted in 259 and 65 subjects in the training and test data. Then RF analysis was applied on the training data, and the resulting output contains the importance table of the miRNAs. The Mean Decrease Accuracy for a predictor measures how much the average prediction accuracy is decreased if the predictor is dropped from the model. Obviously, the predictor with a higher value of mean decrease of accuracy is more important than a lower value in terms of prediction performance. The mean decrease in Gini coefficient is another measure to rank predictors based on their prediction power. A predictor with a higher value of the Mean Decrease Gini represents a higher prediction power, consequently, a predictor with the least mean decrease of Gini possesses the lowest prediction power [23]. The miRNA that appears in the first row of the importance table has the highest importance in prediction of LVI. Similarly, the miRNA appearing in the second row of the table is the second most important in the prediction (Table 3). This importance table revealed that hsa-miR-203a-3p, hsa-miR-30a-3p, hsa-miR-10a-5p, hsa-miR-28-5p, hsa-miR-1266-5p, hsa-miR-187-3p, hsa-miR-584-5p, hsa-miR-194-5p, hsa-miR-30a-5p and hsa-miR-3913-5p are the 10 most important miRNAs. However, due to the random selection of the training set, the output results are altered between individual runs. Hence, to ensure robustness of our data, we have re-analyzed our data five times using the RF method. We record the top 10 important miRNAs from each of the analysis, and found that three miRNAs, hsa-miR-203a-3p, hsa-mir-10a-5p, hsa-miR-194-5p, appear consistently among the 10 most important miRNAs in all 5 analyses. Interestingly all of these 3 miRNAs belong to Wilma cluster 1 as shown in Figure 3.

**Table 3:** Random forest analysis importance Table 5

miRNA name	X0	X1	MeanDecrease Accuracy	MeanDecrease Gini
hsa-miR-203a-3p	13.26	14.30	18.93	7.94
hsa-miR-30a-3p	11.88	5.30	12.16	5.35
hsa-miR-10a-5p	7.10	5.04	8.76	4.48
hsa-miR-28-5p	5.40	6.08	8.15	4.40
hsa-miR-1266-5p	7.43	2.86	7.49	3.56
hsa-miR-187-3p	2.49	5.88	5.81	3.96
hsa-miR-584-5p	8.15	–1.93	5.42	3.19
hsa-miR-194-5p	7.24	–0.59	5.40	3.83
hsa-miR-30a-5p	4.03	3.28	5.26	3.60
hsa-miR-3913-5p	4.91	2.08	5.24	3.71
hsa-miR-1293	5.59	1.09	5.20	3.11
hsa-miR-370-5p	1.01	6.16	4.78	3.64
hsa-miR-101-3p	0.91	6.09	4.77	3.91
hsa-let-7d-5p	3.57	2.96	4.47	3.71
hsa-miR-95-3p	3.63	2.34	4.08	3.28
hsa-miR-181c-3p	5.39	–0.67	4.07	3.15
hsa-miR-455-3p	3.67	1.44	3.74	3.40
hsa-miR-34a-5p	3.26	1.46	3.21	2.73
hsa-miR-9-5p	2.13	2.34	2.97	3.08
hsa-miR-378c	2.89	0.39	2.61	3.46
hsa-miR-128-3p	0.67	2.64	2.19	3.24
hsa-miR-30d-3p	2.31	0.59	2.17	3.11
hsa-miR-1910-5p	2.78	–0.44	1.86	2.61
hsa-miR-141-5p	5.52	–4.55	1.63	2.95
hsa-miR-374a-3p	3.17	–1.81	1.56	2.66
hsa-miR-151a-3p	0.53	1.28	1.14	2.47
hsa-miR-204-5p	0.49	0.55	0.80	2.28
hsa-miR-92b-3p	0.59	0.01	0.59	3.03
hsa-miR-6720-3p	0.37	0.27	0.54	2.63
hsa-miR-106b-5p	1.46	–1.42	0.44	2.40
hsa-miR-181d-5p	0.14	–0.63	–0.20	2.51
hsa-miR-6892-5p	–0.15	–0.47	–0.31	2.36
hsa-miR-194-3p	0.35	–1.73	–0.75	2.35
hsa-let-7f-1-3p	–1.95	–0.40	–1.89	2.45
hsa-miR-1304-3p	–2.85	–0.91	–2.84	2.37

Besides the association between the miRNAs and LVI, we also wanted to figure out the dependence among the miRNAs. For that we calculated partial correlation among the 35 miRNAs selected by the supervised clustering method. The partial correlation between two generic miRNAs, x and y, signifies the correlation between the two after eliminating out the effect of the rest 33 miRNAs. Thus, high partial correlations can be indicators of a possible causal link. It is important to note that partial correlation provides better evidence for regulatory genetic links than the pairwise correlation [24]. Based on the partial correlation matrix we found that hsa-miR-194-5p and hsa-miR-194-3p, hsa-miR-181c-3p and hsa-miR-181d-5p, and hsa-miR-30a-5p are hsa-miR-30a-3p are very highly dependent with the partial correlation of 0.69, 0.72, and 0.88 respectively.

### miRNAs showing increased association with LVI also show increased correlation with lymphangiogenic genes

**Figure 6:**
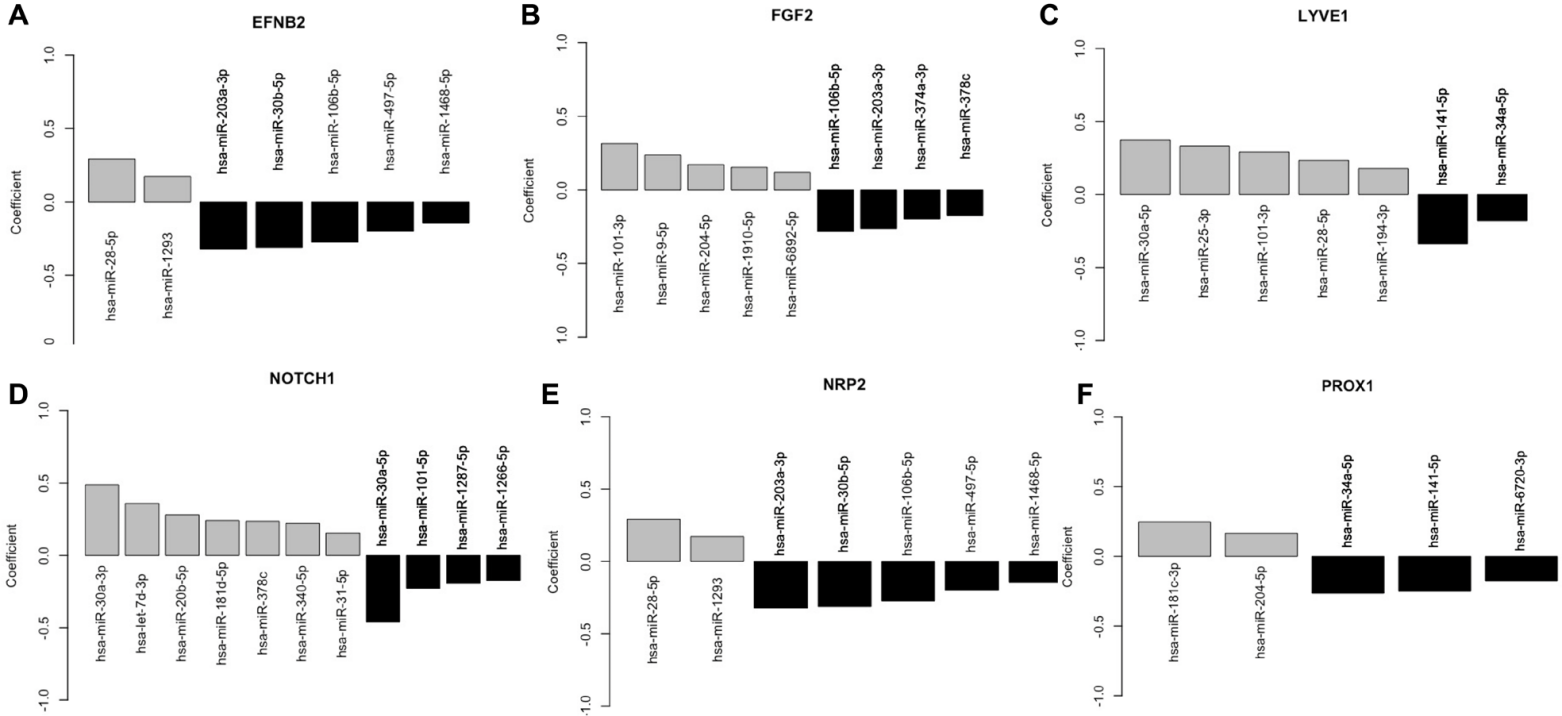
Plot of the regression coefficient of statistically significant miRNAs in the linear regression of the lymphangiogenic genes. The height of the bars represents the estimated regression parameter. Six lymphangiogenic genes EFNB2, FGF2, LYVE1, NOTCH1, NRP2, and PROX1 were considered and regressed its standardized expression on the 57 miRNAs using the multiple linear regression method. (**A**) The top-left plot shows the estimated regression coefficients for the statistically significant miRNAs for EFNB2. Similarly, the other plots of this figure correspond to the genes (**B**) FGF2, (**C**) LYVE1, (**D**) NOTCH1, (**E**) NRP2, and (**F**) PROX1 respectively. Black bars depict a negative association while the grey bars depict a positive association of individual miRNA with the gene.

Since miRNAs directly regulate gene expressions by inhibiting the expression of specific mRNAs, in the next step we wanted to examine if there was any correlation between the 57 shortlisted miRNAs and some of the genes that play an important role in new lymphatic vessel formation or lymphangiogenesis [25] that could be predictive of LVI. To achieve the above-mentioned goal an association study was performed between the selected 57 miRNAs and the following six lymphangiogenic genes, Ephrin B2 (EFNB2), Fibroblast Growth Factor 2 (FGF2), Lymphatic Vessel Endothelial Hyaluronan Receptor 1 (LYVE1), notch receptor 1 (NOTCH1), Neuropilin-2 (NRP2), and Prospero Homeobox 1 (PROX1). To determine the above-mentioned association, we considered each gene and regressed its standardized expression on the 57 miRNAs using the multiple linear regression method. As shown in Figure 6, the top-left plot shows the estimated regression coefficients for the statistically significant miRNAs for EFNB2. Similarly, the other plots of this figure correspond to the genes FGF2, LYVE1, NOTCH1, NRP2, and PROX1 respectively. From this analysis it emerged that hsa-miR-203a-3p has a negative effect on the gene expression values of FGF2 and NRP2. Also, hsa-miR-204-5p has a positive effect on the gene expression values of FGF2 and PROX1. Both hsa-miR-141-5p and hsa-miR-34a-5p have a negative effect on the gene expression values of LYVE1 and PROX1.

### Pathway analysis and target prediction identified several cellular pathways associated with tumor progression and lymph node metastasis

**Figure 7:**
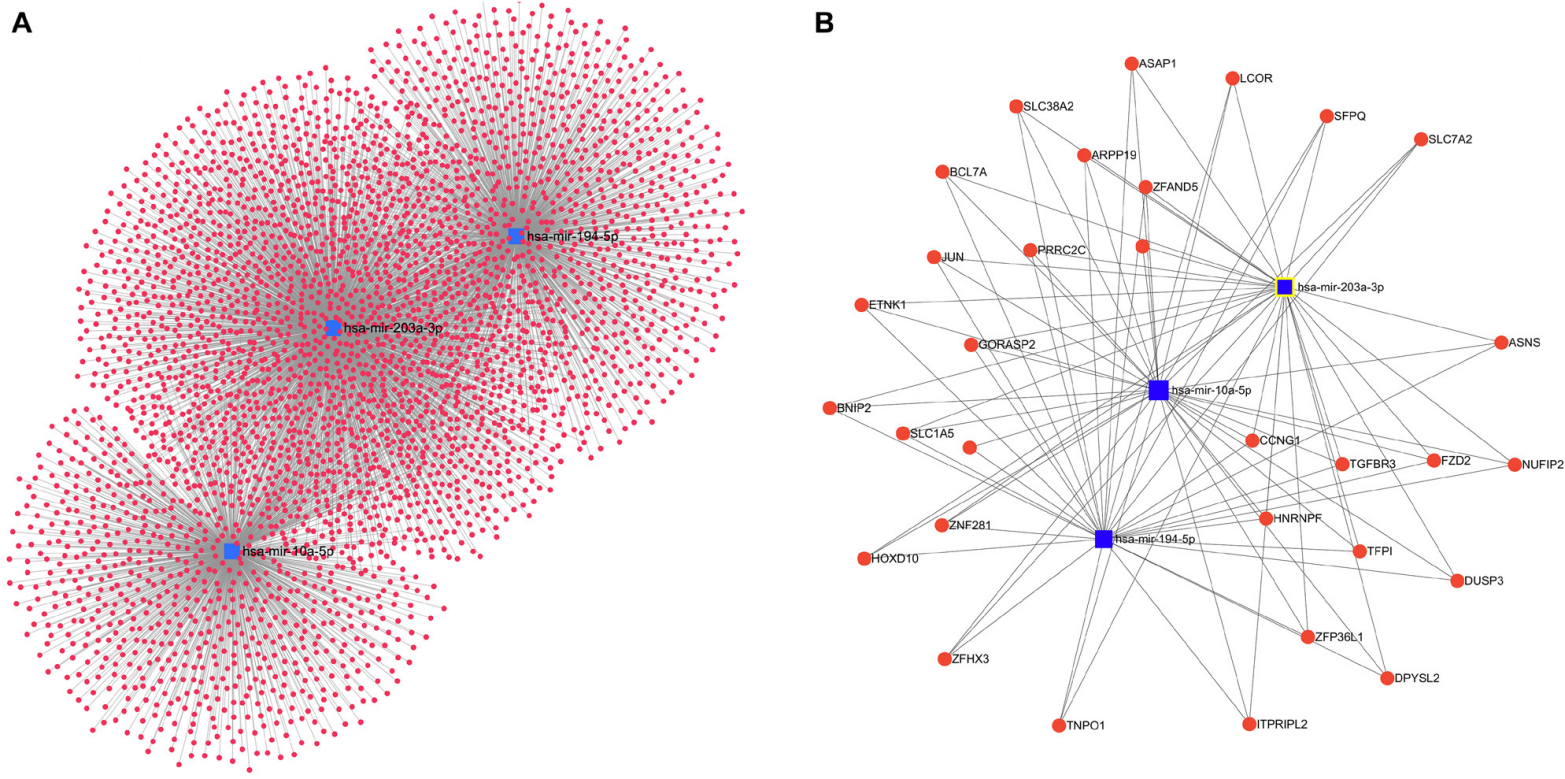
miRNAs and associated interaction with target genes. Network of regulatory genes interacting with miR-194, miR-203a and miR 10a-5p were constructed using miRNET. (**A**) Target genes (3420) interacting with these miRNAs are depicted. (**B**) The minimum gene regulatory network interacting with these three miRNAs are shown. The nodes highlighted in blue represent the miRNAs and the genes are represented by red circles.

The miRNAs in cluster 1 show highest association with LVI and are likely to regulate multiple genes and cellular pathways. Hence pathway analysis was carried out for the miRNAs in Wilma cluster 1 that showed highest association with LVI using the DIANA tools mirPath V3 [26]. The miRNAs that showed a positive correlation with LVI were found to be involved in TGF-β signalling pathway, ECM-receptor interaction, cyclic AMP (cAMP) signaling pathway among the top regulated pathways and the 6 miRNA that has a negative association with LVI in cluster 1 were found to be involved in fatty acid biosynthesis and metabolism, viral carcinogenesis as shown in Supplementary Table 1. Pathway analysis of the two miRNAs hsa-mir-10a-5p, hsa-miR-194-5p that was consistently shown by random forest to be significantly associated with LVI and also identified as positively regulated with LVI in Wilma cluster 1 were found to be involved in regulation of several important cancer associated pathways such as Hippo signaling, adherens junction, fatty acid metabolism, circadian rhythm, TGF-β and p53 signaling pathways (Table 4). Gene target analysis for miR 203a-3p, mir-10a-5p, miR-194-5p was also carried out using the target prediction databases, miRTarBase (https://bio.tools/mirtarbase), and miRDB (http://www.mirdb.org/) [27] and the top 15 target genes for each miRNA showed that the genes are involved in multiple cellular mechanisms (Supplementary Table 2). Further, overlapping interactions of these three miRNAs with multiple genes was also analyzed using miRNet 2.0 (https://www.mirnet.ca) [28] and the total interactive gene regulatory network is shown in Figure 7A. The shortest path network filter was applied to visualize the minimum critical interaction network for these miRNAs and their target genes (Figure 7B).

**Table 4:** Cellular pathways regulated by miR-194-5p and miR-10a-3p

KEGG pathway (Tarbase)	*p*-value	#genes	#miRNAs
Fatty acid biosynthesis	1.73E-23	1	1
Ubiquitin mediated proteolysis	1.26E-06	25	2
Hippo signaling pathway	6.29E-05	18	2
Adherens junction	7.94E-05	13	2
Fatty acid metabolism	0.001	2	2
Circadian rhythm	0.003	9	2
Endocytosis	0.009	24	2
Steroid biosynthesis	0.010	2	2
TGF-beta signaling pathway	0.035	13	2
p53 signaling pathway	0.036	11	2
Bacterial invasion of epithelial cells	0.036	9	2

## DISCUSSION

LVI has been established as an important indicator of disease outcome and treatment modalities in several cancers [8, 29]. In HNSC, LVI is a well-known clinic-pathological feature that tends to adversely influence disease prognosis and negatively affect patient survival and is the primary indicator of lymph node metastasis [11, 13]. Increased lymphangiogenesis is closely related with enhanced LVI and subsequent distant dissemination [10]. Recently, miRNA expression signatures related to prognosis have been found in a number of malignancies [30]. Several miRNAs have been associated with onset, progression and metastasis of HNSC [19, 31]. However, only few studies have evaluated the prognostic significance of dysregulated miRNA expression with increased LNM [32–34]. To the best of our knowledge this is the first study associating the miRNAs that show high correlation with LVI to also be associated with key lymphangiogenic genes. Previous characterization of molecular features in HNSC particularly with the aid of large-scale cancer genomics initiatives such as The Cancer Genome Atlas (TCGA) [14, 35] have generated important insights for stratifying HNSCs and delineating tumor subtypes. However, these studies have typically overlooked association with lymph node or LVI.

It is important to note that metastasis is a complex process involving alterations in several cellular signaling pathways, dysregulation of multiple genes and involves a number of sequential steps not all of which are clearly defined. LVI is an important indicator of metastatic progression and in this study, our primary goal was to delineate the association between miRNA expression and LVI status and identify whether these important miRNAs grouped together in functional clusters that were associated with LVI. The supervised clustering technique is a novel way to achieve both goals. This method allows identification of groups of miRNAs that associate with LVI and likely regulate similar pathways thereby defining biologically relevant pathways that could be targeted to suppress LVI and thereby metastasis.

In our analysis, the preprocessing step obtained by discarding lowly expressed miRNAs followed by step two pruning using the univariate logistic regression to reduce the noise as well as increase the power of detecting significant association. Thus, we obtain the probability of the presence of LVI for a new HNSC cancer patient based on the expression level of their preselected miRNA. Further downstream analysis involved supervised clustering to group miRNAs based on how the miRNAs are affecting the LVI status. The clustering function wilma makes use of the Wilcoxon sign-rank test statistic, adjusted by a well-defined margin function [36]. It is critical to note that Wilma does supervised clustering and clusters only those features that are important with respect to the response, LVI [36]. The unsupervised clustering approach used in this study provided an indication of the optimal number of clusters which was then used in the supervised clustering method that clustered miRNAs incorporating their link with the LVI status. Therefore, our discussion is concentrated on the supervised clusters (Wilma clusters 1, 2, and 3), and the miRNAs within these clusters. Wilma cluster 1 showed the highest predictive power and association with LVI status. The robustness and predictive power of our analyses is revealed by the fact that in addition to the three miRNAs showing the highest correlation with LVI in HNSCC, several of the seventeen miRNAs identified in cluster 1 of the Wilma clustering have been previously associated with HNSCC [37]. For example, miR-30a has been associated as a tumor suppressor in HNSCC [38], high let-7d influences HNSCC growth [39], miR-3913 and miR-6892 have been associated with immune signature and candidate prognostic marker in HNSCC [40], miR-181d is shown to be predictive of risk in HNSCC [41], miR-141 suppresses growth and metastatic potential [42]; miR-370 has been shown to regulate insulin sensitivity and modulate tumor growth [43], miR-378 [44], miR-1910 and miR-187 are shown to be induced in HNSCC [10, 45]. However, we also noted that three of the miRNAs, miR-6720, miR-584, and miR-1304-3p have not been shown to be associated with HNSCC as a prognostic indicator. This is an interesting finding as it also underscores the power of these predictive models to identify novel miRNAs that are associated with LVI in HNSCC.

Hence, for the purpose of analyzing the cellular pathways affected we focused on that cluster. According to the functional pathway analysis of the miRNAs in Wilma cluster 1, we found that the target genes of these miRNAs that were positively associated with LVI were associated with several important tumor enhancing processes, including TGF-β signaling pathway and ECM-receptor interaction mechanisms. This is significant as the TGF-β signaling pathway plays an important role in tumor metastasis and is involved in mediating early lymph node metastasis in multiple cancers [46, 47]. TGF-β exerts a complex dual regulation of the lymphangiogenic processes [48]. Tumor cells, can promote tumor lymphangiogenesis and lymph node metastasis by activating mechanisms through TGF-β to increase the expression of lymphangiogenic molecules as Vascular Endothelial Growth Factor C (VEGF-C) [49] while direct effects of TGF-β on lymphangiogenesis is inhibitory [49]. Alteration in the extracellular matrix components significantly modulate the tumor microenvironment and contributes to metastasis and has been associated with increased LVI in several other cancers [50, 51]. Our heatmap also pointed to 3 main clusters of the miRNAs.

Using RF approach, we identified several miRNAs that strongly correlated to LVI. Interestingly, almost all of the miRNAs that were identified by our random forest analysis to be significantly associated with LVI have also been independently identified in a number of studies in HNSC giving further credibility to our RF model. We focus our discussion here on the three miRNA (miR-203a-3p, hsa-miR-194-5p, miR-10a-5p,) that were consistently identified by the multiple iterations of RF analysis and also belong to Wilma cluster 1 that shows highest predictive association with LVI. These miRNAs were chosen based on the fact that they were validated as the highest predictors across multiple analyses. We want to point out that our main three important miRNAs hsa-miR-203a-3p, hsa-mir-10a-5p, hsa-miR-194-5p fell in two different clusters based on the heatmap- hsa-mir-203-3p and hsa-mir-10a-5p in one cluster and hsa-mir-194-5p in other. This is not a surprise as the heatmap (for hierarchical clustering) did unsupervised clustering whereas Wilma provided supervised clustering. Secondly, the heatmap was based on the unscaled expression values whereas the k-means clustering and the Wilma clustering were applied on the scaled expression values. Our analysis showed that miR-203a-3p was negatively associated with LVI. This fits well with other studies where miR-203a-3p has been reported as a tumor suppressor and dysregulated in many malignancies including nasopharyngeal carcinoma [52], gastric cancer [53] and bladder cancer [54]. miR-203 has also been shown to be downregulated in animal models of oral cancer-a subtype of HNSCC [37]. However, it also functions as an oncomiR in breast cancer [55] and hepatocellular cancers [56] depending on the genes targeted. Significantly, miR-203a-3p suppresses expression of SOCS3 that has been shown to be closely associated with lymph node metastasis in breast cancers [57]. One of the predicted targets of miR-203a in our analysis was Semaphorin 5a that is associated with lymph node metastasis and adverse prognosis [58]. Integrative Analysis of miRNAs in HNSCC has previously identified miR-194 to be associated with the epithelial sub-type of HNSCC [59]. However, to the best of our knowledge no study has shown the role of miR-203, miR-194 and miR-10a as a prognostic indicator of LVI in HNSCC. miR-194 has been proved as a tumor promoting factor in various cancers and regulates the EMT mechanisms promoting cancer growth [60]. It has been reported to be significantly elevated in lymph node metastatic tissues from colorectal cancer patients [60]. miR-10a-5p has been reported to be overexpressed and to act as an important mediator of metastasis formation in PDAC [61]. Aberrant expression of miR-10a has been reported in head and neck cancers [62] and increased expression of miR-10a-5p has been shown to be associated with clinicopathological characteristics such as age and gender in laryngeal cancer [63]. In gastric cancer, miR-10a has been shown to have an important role in the metastasis from primary GC to lymph nodes [64]. Also, one of the top 20 miRNAs that showed strong correlation with survival as well as showed a significant positive correlation with LVI was miR-9 that we have previously demonstrated to be an important regulator of lymphangiogenesis and lymphatic tube formation, which is the first step to promote tumor spread through lymphatics [65]. Further, overexpression of miR-9 has been associated with poor prognosis in several cancers [66] and it has also been associated with increased lymph node metastasis in breast cancers [67]. KEGG pathway analysis of miR-194 and miR-10 which are positively corelated with LVI, revealed several pathways such as Fatty Acid synthesis, Hippo signaling, p53 pathways, TGF-β pathways and others. Aberrant production of fatty acids is associated with poor prognosis in human cancers and inhibition of this pathway has been associated with decreased LNM [68]. The Hippo signaling pathway is particularly significant as it is involved in control of tumorigenesis, and has recently been linked to metabolic reprogramming in metastatic lymph nodes [69–71]. Thus, regulation of these pathways could have significant impact on LVI and subsequent lymph node metastasis and patient outcome. In one study, weighted gene co-expression network analysis was used to construct gene co-expression networks and investigate the relationship between key modules and the LVI clinical phenotype and identified 24 genes in the metabolic and immune reprogramming [13]. This is interesting all the miRNAs (miR-194, miR-10a and miR 203a) that show significant association with LVI in our study also regulate metabolic pathways and alteration of immune cell response that are known to remodel the metastatic lymph node [72–74].

Tumor cells express high amounts of angiogenic molecules (that promote growth of new blood vessels) or lymphangiogenic molecules (that promote growth of new lymphatic vessels) that are critical for metastasis [75]. However, existing work in this area often does not take into account the dynamics of these interactions between miRNAs and regulators of angiogenic or lymphangiogenic pathways that promote lymph node invasion and hence makes therapeutic targeting of metastatic cancers a critical challenge. Our miRNA correlation data with lymphangiogenic genes corroborates with other independent studies with miRNA target validation in literature. FGF2 has been validated as a direct target of miR-203. It has been shown that miR 203 inhibits renal cancer cell proliferation, migration and invasion by targeting of FGF2 [76]. Although studies have not directly established LYVE1 as a target for either miR-141 or miR-34a, increased LYVE1 expression has been shown to be associated with increased rate of LNM in oral cancer [77]. Hence therapeutic targeting of LYVE 1 may suppress LNM in oral and HNSC. Both miR-141 and miR-34 are actively involved in regulation of epithelial-mesenchymal transition (EMT) mechanisms and recent studies suggest that LYVE and PROX1 are also involved in EMT that promotes LNM [78]. The association of miR-141 with both LYVE1 and PROX1 could potentially be a novel axis unraveled by our analysis that could provide new mechanistic links in EMT pathways in LNM progression. However, further studies are warranted. Our data shows that specific miRNAs (i.e., miR-141, miR-34a) show a negative association with two key lymphangiogenic genes PROX1 and LYVE. On the other hand, some specific miRNAs show positive association with LYVE1 (miR-30a, miR-25, miR-101) vs PROX1 (miR-181, miR-204). These are critical findings as it suggests that these miRNAs suppress expression of genes that maybe novel regulators that could positively or negatively regulate these lymphangiogenic genes in a context dependent manner. Interestingly, miR-203 expression was negatively associated with three of the six lymphangiogenic genes studied (EFNB2, NRP2 and FGF) suggesting that downregulation of miR-203 potentiates lymphangiogenic mechanisms. Further, our data showed that miR-204-5p is positively co-related with FGF-2 and PROX1. It has been shown that miR-204-5p promotes tumor angiogenesis through regulation of thrombospondin 1 [79] and similar mechanisms could be at play in the regulation of lymphangiogenesis. Further analysis is warranted to identify some of these mechanisms and could lead to some novel new targets.

In conclusion, our results showed that specific miRNA clusters significantly associate with LVI. The identified miRNA clusters regulate multiple biological pathways that are involved in progression of LNM and could be potential predictors of metastatic disease. In addition, RF analysis revealed three miRNAs hsa-miR-203a-3p, hsa-mir-10a-5p, hsa-miR-194-5p to be most strongly associated with LVI and can be used as important prognostic indicators. Further, we also find that each of these miRNAs have association with significant molecular pathways as cell proliferation, metabolism and lymphangiogenesis that further promotes tumor progression. In addition, all of the identified miRNAs are associated with metabolic pathways as well as immune cell responses that define alterations in a metastatic lymph node so it also potentiates development of specific targeted interventions to these pathways that may distinguish a metastatic from a naïve lymph node. Different miRNAs regulate different aspects of metastatic progression and identification of specific miRNA linked with LVI, lymphangiogenesis and subsequent nodal metastasis may help in identification of patients at early stages of disease progression. Specific miRNA agonists (mimics) or antagonists (inhibitors) can be evaluated further in pre-clinical trials to evaluate their efficacy in inhibition of HNSCC progression when detected in earlier stages. Further studies are warranted to determine the functional relevance of these findings and evaluate the downstream targets in tumor models of HNSCC metastasis.

## MATERIALS AND METHODS

### TCGA data retrieval

**Figure 8:**
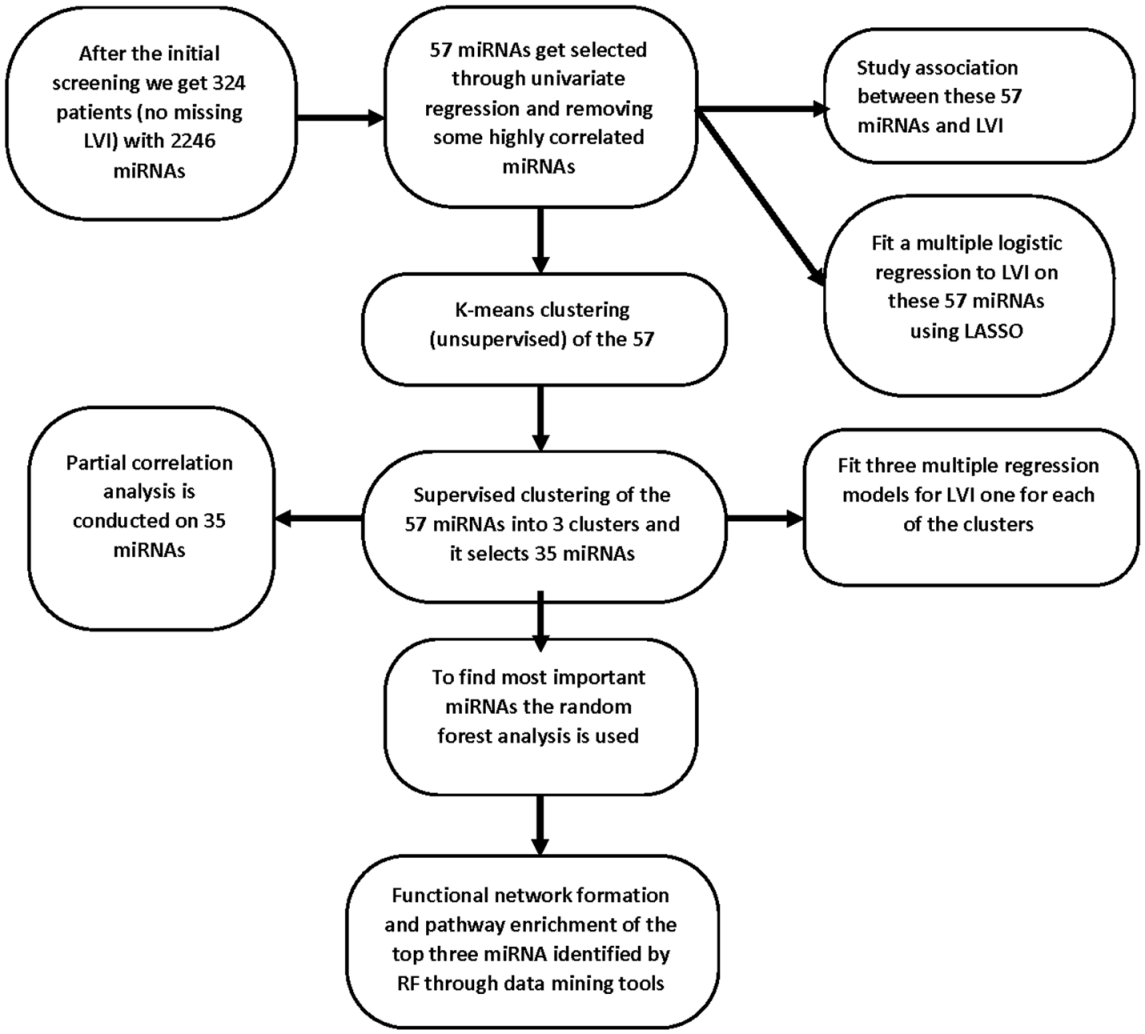
Flow chart showing the overall workflow describing the steps used to develop the prognostic model and identification of miRNAs that are predictive of LVI. Abbreviations: TCGA: The Cancer Genome Atlas; HNSCC: head and neck squamous cell carcinoma; OS: overall survival; ROC: receiver operating characteristic; LASSO: least absolute shrinkage and selection operator regression.

We used the Head and Neck squamous cell carcinoma (HNSC) cohort of the TCGA data portal. The overall steps of the workflow are depicted in Figure 8. To download and integrate the clinical and genomic data from TCGA we have used the web-based platform, University of California Santa Cruz Xena Browser (https://xenabrowser.net/). We obtained mature miRNA sequences (IlluminaHiseq) referenced with a miMAT accession number. Particularly, the sequences are level-3 data, and log2(total RPM+1), where total RPM represents the total of all isoform expressions for the same miRNA mature strand. The clinical data on subjects are also obtained through UCSC Xena, with the major focus on lymphovascular invasion status among many other phenotypic variables and in the downloaded data it was named as lymphovascular_invasion_present. This variable is referred to as LVI. Although the clinical data had information on 604 subjects, after removing subjects without any LVI information we were left with only 411 patients. Matching the miRNA sequence data with their corresponding clinical data, we obtained a total of 351 patients. The TCGA consortium contains data from five different sample types [80]. Out of these patients’ samples, we considered only the primary solid tumor tissue, and that left us with 324 patients. After the analysis, we retrieved the information of mature miRNA corresponding to the miMAT accession number using the R package miRBaseConverter available from https://www.bioconductor.org [81]. The HNSC data contains expression data of 2246 miRNAs. The variable of interest LVI is a binary variable. Value zero indicates that the tumor cell doesn’t invade through the lymphatic vessels yet while value one indicates the invasion of cancer to lymphatic vessels. Among 324 patients, 211 patients did not have LVI (LVI) while 113 patients show LVI. Table 5 summarizes clinical information including gender, age, race, LVI, clinical stages, alcohol and tobacco smoking history, and anatomical site information of the patients. Association of the demographic and clinical information with LVI was also calculated.

**Table 5:** Clinico-pathological features of HNSC patients grouped by the lymphovascular invasion status (LVI) in TCGA study cohort

		LVI- No (211)	LVI-Yes (113)	*p*-value
Gender	Female	68	23	0.033
	Male	143	90	
Age of initial diagnosis	Minimum	24 years	26 years	0.061
	Maximum	87 years	87 years	
	Median	61 years	59 years	
	Average	61.5 years	59.1 years	
Alcohol history documented	No	70	31	0.304
	Yes	136	81	
	NA	5	1	
Alcohol consumption per day	0	34	15	0.001
	1	10	11	
	(1, 3)	22	11	
	more than 3	25	22	
	NA	120	54	
Tobacco smoking history indicator	1	60	22	0.002
	2	61	44	
	3	33	8	
	4	51	35	
	5	1	1	
	NA	5	3	
Clinical stage	Stage I	12	4	0.109
	Stage II	46	10	
	Stage III	51	22	
	Stage IVA	89	72	
	Stage IVB	4	1	
	Stage IVC	2	0	
	NA	7	4	
Anatomical neoplasm subdivision	Alveolar ridge	9	5	0.119
	Base of tongue	6	5	
	Buccal mucosa	10	6	
	Floor of mouth	32	18	
	Hard palate	4	0	
	Hypopharynx	3	1	
	Larynx	35	34	
	Lip	1	0	
	Oral cavity	29	10	
	Oral tongue	70	25	
	Oropharynx	4	2	
	Tonsil	8	7	

^#^The numerals used in tobacco smoking history signify the following: 1- Lifelong Non-smoker (less than 100 cigarettes smoked in Lifetime), 2-Current smoker (daily smokers, non-daily smokers or occasionalsmokers), 3-Current reformed smoker for > 15 years (greater than 15 years), 4-Current reformed smoker for ≤15 years, 5-Current reformed smoker, duration not specified, NA-Smoking History not documented.

### Statistical analysis and preprocessing of miRNA

For the statistical analyses, R software, version 3.6.2 was used. As a first step of pre-processing the data, we only considered miRNAs whose mean value across the patients were greater than one to avoid miRNAs with very low expression. After this exclusion, 496 miRNAs fit the criteria for further association studies. Finally, we standardized each miRNA expression to have zero mean and unit variance. Standardized values of every miRNA were obtained by subtracting the mean and then dividing by the standard deviation of the respective miRNA. The primary goal of our analysis was to identify the important miRNAs based on their association with LVI, or predictive power of the LVI status using statistical methods, and investigate their biological role in this context. At the very first stage, instead of working with all available miRNAs, we performed a second level of preprocessing to preselect a subset of miRNAs that are significantly associated with the binary LVI status based on the univariate logistic regression model. Specifically, we used the glm function to run the logistic regression. Based on the 5% significance level, we screened out important miRNAs that will be used for downstream analyses. Next, we computed the pairwise correlation among the miRNAs, and removed a handful of miRNAs that are highly correlated with others. Then, to find a best predictive model we fitted a multiple logistic regression model to LVI on all the miRNAs that were selected in the previous step. Since a large number of miRNAs are included simultaneously as predictors we used the penalized estimation method, the least absolute shrinkage and selection operator (LASSO) technique [21], to select the important miRNAs for the best predictive model.

### Supervised and unsupervised clustering of miRNA

**Figure 9:**
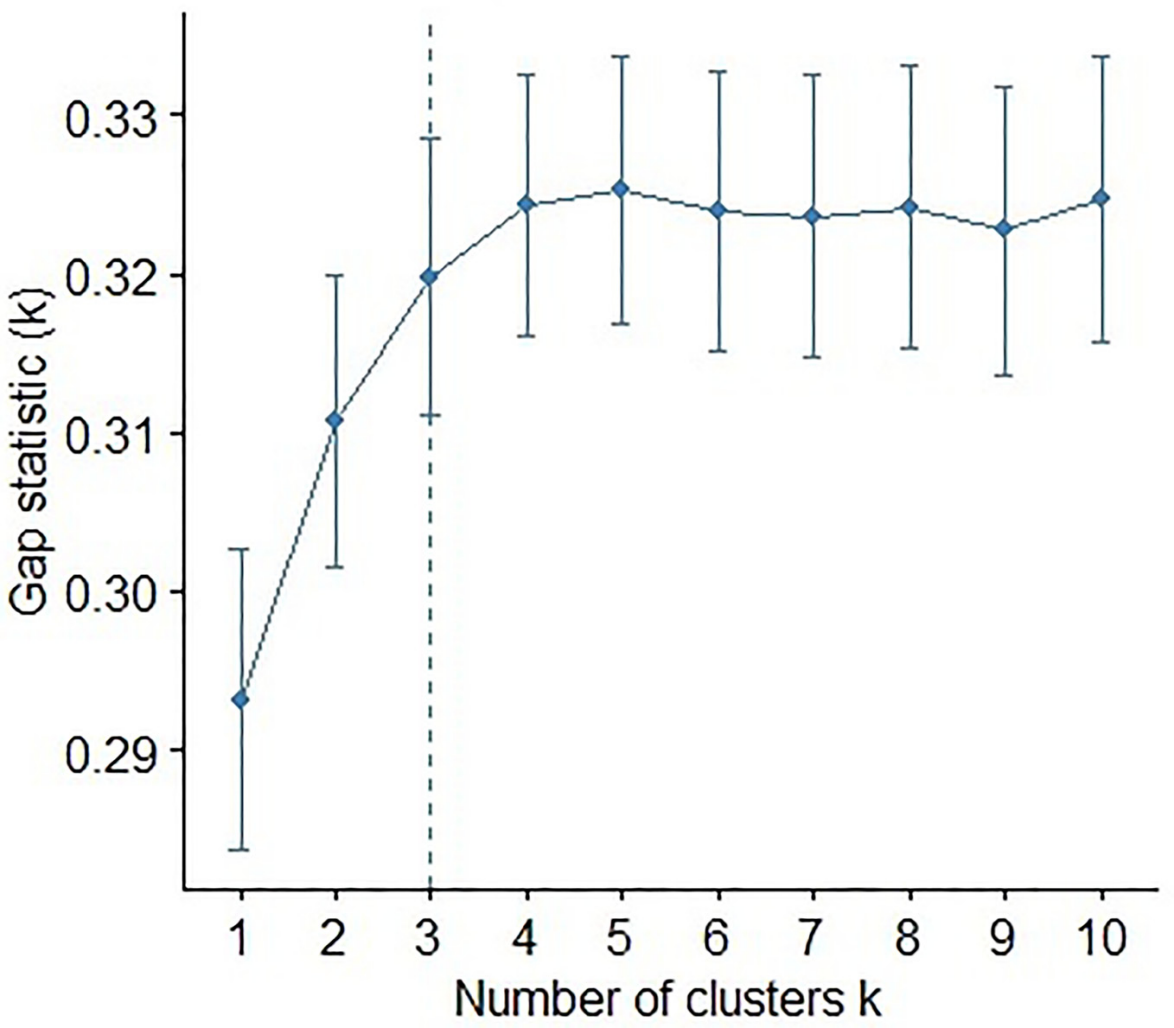
Plot of the gap statistic against the number of clusters in an unsupervised k-means clustering of the miRNAs. Bars at every point represent the +/– 1 standard error of the gap statistic.

Besides finding a good predictive model, we wanted to examine if these miRNAs would show more connection with others and show functional clusters that are indicative of increased risk for LVI. Hence, clustering of miRNAs is a novel component of our analyses. Most of the supervised techniques rely on prior knowledge on the number of clusters [82]. Therefore, we first implement unsupervised K-means clustering with the gap statistic (using the clusGap function) to determine the optimal number of clusters. K-means uses a distance measurement to calculate the similarity between miRNAs. The gap statistic compares the total within-cluster variation for different values of *k* with their expected values under a null reference distribution of the data. The reference dataset is generated using Monte Carlo simulations of the sampling process. Figure 9 shows the gap statistic corresponding to different values of the number of clusters. Next, we used the Wilma function of the supclust package of R [83] to do supervised clustering using the miRNAs that were selected based on the 5% significance level of the univariate logistic regression and after eliminating the miRNAs that were highly correlated with others. The clustering function Wilma makes use of the Wilcoxon sign-rank test statistic, adjusted by a well-defined margin function to implement the above-mentioned algorithm [36]. The optimal number of clusters of the unsupervised method was used in the Wilma function. We used Bioconductor package supclust to perform supervised clustering [36]. The mentioned package needs a user-specified cluster number that was three in our case as indicated by K-means clustering with the Gap statistic as shown in Figure 9. The supervised algorithm tries to find miRNAs clusters, so that the average expression profile of each cluster has a great potential for explaining the LVI status. At the initial step, the algorithm starts out with the best single miRNAs in explaining invasion status and then adds one after another to the existing cluster. We then compared the predictive power of each of the Wilma clusters of miRNAs through the multiple logistic regression analysis and the ROC curve. Following the request of a reviewer, a hierarchical clustering of the non-standardized log2 transformed miRNAs expression in correlation to LVI status was created. For this we used the pheatmap function of R Package Version 1.0.12. (https://CRAN.R-project.org/package=pheatmap) [84].

### Random forest analysis

To determine the most important miRNAs from the set of miRNAs identified by the Wilma cluster, we performed a random forest (RF) analysis [22]. To identify which of the miRNAs have prognostic value, in the RF analysis, we used LVI as the binary response and implemented a machine learning strategy. RF orders miRNAs according to their importance in predicting the LVI status based on a decision-tree based approach. For a RF analysis of a dataset, first the observations of the dataset are randomly divided into two parts, such as 80% and 20%. The larger portion is then used as a training dataset and the smaller set is used as a test dataset. The training data are used to tune the decision tree-based algorithm. The test data are used for checking the predictive capability of the algorithm. The RF and the multiple logistic regression based on the Least absolute shrinkage and selection operator (LASSO) penalty are analogous approaches as both are used for identifying predictive miRNAs, but one is model based and the other is model-free approach.

## SUPPLEMENTARY MATERIALS



## Abbreviations

HNSC: Head and neck cancers
miRNAs: microRNAs
TCGA: The Cancer Genome Atlas
LVI: Lymphovascular Invasion
Lasso: Least absolute shrinkage and selection operator
RF: Random forest
EFNB2: Ephrin B2
FGF2: Fibroblast Growth Factor 2
LYVE1: Lymphatic Vessel Endothelial Hyaluronan Receptor 1
NOTCH1: notch receptor 1
NRP2: Neuropilin-2
PROX1: Prospero Homeobox 1
cAMP: cyclic AMP
LNM: lymph node metastasis
EMT: epithelial-mesenchymal transition
