# Supplementary material for: Identification of miR-203a, mir-10a, and miR-194 as predictors for risk of lymphovascular invasion in head and neck cancers
Source: Oncotarget. 2021 Jul 20;12(15):1499–519. doi: 10.18632/oncotarget.28022 (PMC8310671; doi:10.18632/oncotarget.28022)
Supplement: Supplementary file 1 [file oncotarget-12-1499-s001.pdf]

Identification of miR-203a, mir-10a, and miR-194 as predictors for risk of lymphovascular invasion in head and neck cancers

SUPPLEMENTARY MATERIALS

Supplementary Table 1: Cellular pathways regulated by miRNAs positively associated with LVI in Wilma Cluster 1

**miRNAs positively associated with LVI**<br>**KEGG pathway (Tarbase)**	***p*-value**	**#genes**	**#miRNAs**
Protein processing	2.10E-09	83	8
Proteoglycans in cancer	2.10E-09	91	8
Prion diseases	2.40E-09	11	6
Viral carcinogenesis	1.76E-08	83	8
Fatty acid biosynthesis	1.12E-07	5	4
Adherens junction	2.56E-07	41	7
Oocyte meiosis	7.30E-07	53	7
Ubiquitin mediated proteolysis	1.22E-06	68	7
Hippo signaling pathway	1.66E-06	60	8
Pathways in cancer	1.88E-06	152	8
Cell cycle	4.94E-06	60	8
Lysine degradation	7.40E-06	22	8
TGF-beta signaling pathway	2.88E-05	37	7
Renal cell carcinoma	5.74E-05	33	8
RNA transport	0.000	71	7
mTOR signaling pathway	0.000	32	8
Prostate cancer	0.001	41	8
Spliceosome	0.001	61	7
Apoptosis	0.001	37	8
**miRNAs negatively associated with LVI**<br>**KEGG pathway (Tarbase)**	***p*-value**	**#genes**	**#miRNAs**
Prion diseases	3.30E-36	1	1
Fatty acid biosynthesis	1.63E-33	1	1
Fatty acid metabolism	9.71E-21	4	2
Huntington’s disease	1.06E-06	11	3
Biosynthesis of unsaturated fatty acids	0.000	2	1
Viral carcinogenesis	0.000	14	3
Spliceosome	0.021	13	2
Estrogen signaling pathway	0.021	8	2
Glycosphingolipid biosynthesis - ganglio series	0.046	1	1

Supplementary Table 2: Predicted gene targets for miR-203a-3p, miR-194-3p and miR-10a-5p as identified from miRDB

miRNA	Targets from miRDB	Gene name
hsa-miR-203a-3p	ELL2	elongation factor for RNA polymerase II 2
hsa-miR-203a-3p	ADAMTS6	ADAM metallopeptidase with thrombospondin type 1 motif 6
hsa-miR-203a-3p	AFF4	AF4/FMR2 family member 4
hsa-miR-203a-3p	PHIP	pleckstrin homology domain interacting protein
hsa-miR-203a-3p	CAB39	calcium binding protein 39
hsa-miR-203a-3p	THSD7A	thrombospondin type 1 domain containing 7A
hsa-miR-203a-3p	BBX	BBX, HMG-box containing
hsa-miR-203a-3p	PDE4D	phosphodiesterase 4D
hsa-miR-203a-3p	CASK	calcium/calmodulin dependent serine protein kinase
hsa-miR-203a-3p	GUCY1A2	guanylate cyclase 1 soluble subunit alpha 2
hsa-miR-203a-3p	SEMA5A	semaphorin 5A
hsa-miR-203a-3p	TMEM154	transmembrane protein 154
hsa-miR-203a-3p	MEX3C	mex-3 RNA binding family member C
hsa-miR-203a-3p	ELL2	elongation factor for RNA polymerase II 2
hsa-miR-203a-3p	ADAMTS6	ADAM metallopeptidase with thrombospondin type 1motif 6
hsa-miR-194-3p	FBXW7	F-box and WD repeat domain containing 7
hsa-miR-194-3p	GNPTAB	N-acetylglucosamine-1-phosphate transferase subunits
hsa-miR-194-3p	SZRD1	SUZ RNA binding domain containing 1
hsa-miR-194-3p	TAB2	TGF-beta activated kinase 1 (MAP3K7) binding protein 2
hsa-miR-194-3p	KCNAB2	potassium voltage-gated channel subfamily A
hsa-miR-194-3p	EID1	EP300 interacting inhibitor of differentiation 1
hsa-miR-194-3p	LEPROT	leptin receptor overlapping transcript
hsa-miR-194-3p	DEPDC1	DEP domain containing 1
hsa-miR-194-3p	NRM	nurim
hsa-miR-194-3p	GJB4	gap junction protein beta 4
hsa-miR-194-3p	RAP1GDS1	Rap1 GTPase-GDP dissociation stimulator 1
hsa-miR-194-3p	YWHAE	tyrosine 3-monooxygenase activation protein epsilon
hsa-miR-194-3p	LPGAT1	lysophosphatidylglycerol acyltransferase 1
hsa-miR-194-3p	NACC2	NACC family member 2
hsa-miR-194-3p	RASL12	RAS like family 12
hsa-miR-10a-5p	CADM2	cell adhesion molecule 2
hsa-miR-10a-5p	TFAP2C	transcription factor AP-2 gamma
hsa-miR-10a-5p	CNOT6	CCR4-NOT transcription complex subunit 6
hsa-miR-10a-5p	RORA	RAR related orphan receptor A
hsa-miR-10a-5p	GALNT1	polypeptide N-acetylgalactosaminyltransferase 1
hsa-miR-10a-5p	E2F7	E2F transcription factor 7
hsa-miR-10a-5p	CRLF3	cytokine receptor like factor 3
hsa-miR-10a-5p	KCNA6	potassium voltage-gated channel subfamily A member 6
hsa-miR-10a-5p	SOBP	sine oculis binding protein homolog
hsa-miR-10a-5p	ELOVL2	ELOVL fatty acid elongase 2
hsa-miR-10a-5p	NR6A1	nuclear receptor subfamily 6 group A member 1
hsa-miR-10a-5p	CDK6	cyclin dependent kinase 6
hsa-miR-10a-5p	SIX4	SIX homeobox 4
hsa-miR-10a-5p	EBF2	EBF transcription factor 2
